# Neural activation is enhanced with operational task ecological validity during complex cognitive tasks

**DOI:** 10.3389/fnhum.2026.1825048

**Published:** 2026-05-21

**Authors:** Luca E. Bonarrigo, Iris Li, Daniel C. Comstock, Karen M. Baldonado, Wyatt Rees, Torin K. Clark, Lee M. Miller, Eric A. Vance, Stephen Robinson, Daniel Szafir, Allison P. Hayman

**Affiliations:** 1 Bioastronautics Laboratory, Ann & H.J. Smead Aerospace Engineering Sciences, University of Colorado Boulder, Boulder, CO, United States; 2 Department of Neurobiology, Physiology, & Behavior, Department of Otolaryngology, Head and Neck Surgery, and Center for Mind & Brain, University of California, Davis, Davis, CA, United States; 3 Department of Mechanical and Aerospace Engineering, University of California, Davis, Davis, CA United States; 4 Department of Applied Mathematics, University of Colorado Boulder, Boulder, CO, United States; 5 Department of Computer Science, University of North Carolina at Chapel Hill, Chapel Hill, NC, United States

**Keywords:** cognitive decrements, EEG, fNIRS, spaceflight, virtual reality

## Abstract

**Introduction:**

Extended exposure to a microgravity environment has been related to cognitive and neural decrements in astronauts, including changes in brain morphology and connectivity. Future long-duration exploration missions, such as those to Mars, will require the development of new countermeasures to counteract these decrements. Training in virtual reality (VR) has been identified as a promising potential countermeasure. Though there has been extensive research into VR as a tool for neurorehabilitation and microcognitive testing, little is understood about the neural effects of training in VR for operationally relevant tasks.

**Methods:**

This research utilized functional near infrared spectroscopy (fNIRS) and electroencepholography (EEG) to measure neural activation during task completion in a VR environment with spaceflight-relevant tasks as compared to one with microcognitive corollary tasks.

**Results:**

We find that a complex, operationally relevant VR environment elicits enhanced brain activation compared to the matching corollary tasks designed to target equivalent specific cognitive domains, for both EEG (*p* < 0.0005) and fNIRS (*p* < 0.001).

**Discussion:**

These results indicate that brain activation and recruitment is increased when the task has higher ecological validity, providing an objective assessment to inform countermeasure development for spaceflight associated neural decrements.

## 1 Introduction

The next era of human space exploration will require much longer and farther-reaching missions to the moon and Mars. With long duration exploration missions (LDEMs) come many challenges, one of which is maintaining astronaut skills for complex operational tasks such as driving a rover, operating a robotic arm, and technical maintenance (Barshi and Dempsey, 2016). These skills require the astronauts to maintain cognitive and sensorimotor function over the course of years to ensure mission success.

Long-duration spaceflight can induce changes in astronauts' brain morphology and connectivity. Specifically, previous research has identified a decrease in gray matter in frontal and temporal lobes and increase in the sensorimotor regions (Koppelmans et al., 2016; Van Ombergen et al., 2018), as well as changes in cerebrospinal fluid distribution, an increase in white matter in motor regions, and an upward shift of the brain (Jillings et al., 2020; Roberts et al., 2017). Further, space radiation has the potential to lead to degradation of the central nervous system (Gauger et al., 1986; Straume, 2018).

Despite extensive evidence of spaceflight-induced structural changes to the brain, how this translates to cognitive performance decrements during spaceflight is poorly understood. Psychological stressors during spaceflight such as circadian disruption, isolation, personal stress, fatigue and confinement all negatively affect cognitive and behavioral health (Clément et al., 2020; Roy-O'Reilly et al., 2021). Astronauts on long-duration missions commonly report experiencing “space fog,” a term used to describe the feelings of disorientation, reduced cognitive ability, mental dullness, difficulty concentrating, and mental fatigue that astronauts commonly experience in orbit (Clément et al., 2020), but changes in cognitive performance as measured by simplified microcognitive tasks, including the WINSCAT used on the International Space Station (ISS), have yielded widely variable results across individuals (Roberts et al., 2019; Strangman et al., 2014; Tays et al., 2021). More recent longitudinal research measuring cognitive performance of astronauts aboard the ISS with the Cognition Test Battery (CTB) yielded no evidence for a systematic cognitive performance decline over a 6-month mission, though differences from pre-flight baseline varied very widely from individual to individual and across different phases of spaceflight (Dev et al., 2024). While the results on spaceflight's effects on cognitive performance are largely inconclusive, research has shown reductions in sensory integration and processing after long periods in microgravity, as well as motor function impairments, which are both critical to mission success (Clark, 2019; Tays et al., 2021; Van Ombergen et al., 2017). Regardless of the causes, however, the complexity of tasks that astronauts are required to complete on their missions, from elaborate research procedures to spacewalks, require astronauts to maintain a high level of both cognitive and motor/physical performance to avoid errors that may have devastating impacts to task outcomes and safety.

The exact mechanisms and contributing factors behind the complex and highly individualistic neuropsychological changes during spaceflight are also not well understood. LDEMs such as missions to Mars will require the development of new countermeasures to counteract these neural decrements. Current neurological countermeasures on the ISS are limited to behavioral health mitigations and psychiatric pharmaceutical medications (Friedman and Bui, 2017; Gatti et al., 2022), and although recent work has aimed to characterize the utility of non-pharmaceutical interventions such as transcranial brain stimulation (Faerman et al., 2024; Roberts et al., 2010), these potential countermeasures have yet to be deployed in space. While microcognitive-based assessments such as the WINSCAT and CTB have been used to characterize cognitive changes during spaceflight (Basner et al., 2015; Kane et al., 2005), these tasks are regarded to be too simplified to capture the macrocognitive processes associated with the complex tasks astronauts complete daily (Chen et al., 2019). Thus, there is a need for new countermeasures to fill this gap.

Virtual reality (VR) has been identified as a promising potential countermeasure to many clinical neurological changes. VR has been successfully shown to improve effectiveness of neurorehabilitation on Earth for patients with cerebral palsy and stroke, improve functional connectivity between affected regions of the brain, and enhance sensorimotor learning by stimulating neural activity through immersive visual modalities (Chen et al., 2007; Hao et al., 2022; McEwen et al., 2014). Further, EEG studies have suggested that VR can engage multiple cortical regions associated with motor systems and improve motor performance (Massetti et al., 2018) and neuroplasticity to enhance cognitive rehabilitation (Gangemi et al., 2023). Thus, VR may be a promising countermeasure to the potential detrimental neurological effects of microgravity.

VR may also serve as a form of in-transit training for LDEMs to maintain the complex skills that were trained on ground pre-flight. VR has been effective at training for scenarios that require the integration of multiple cognitive tasks and are conducted in high-risk environments, including but not limited to surgical procedures, firefighting, and operational maintenance tasks (Aïm et al., 2016; Crison et al., 2005; Engelbrecht et al., 2019; Ganier et al., 2014; Satava, 1993). NASA currently uses VR training for astronauts in ground analogs prior to their spaceflights as well as onboard the ISS (Garcia et al., 2020; Rycroft et al., 1999). Thus, the operational relevance of VR adds further value to its potential as a neurological countermeasure.

Given this potential, it is necessary to investigate the details of how a high-quality, immersive, and operationally relevant VR training environment stimulates the brain as compared to the lower-quality, non-immersive, simplified cognitive tasks that target the same regions of the brain. Thus, microcognitive tasks similar to those used within the CTB or WINSCAT on the ISS can serve as a reference against which to compare cortical engagement in various cognitive domains of interest.

To inform a gap in knowledge and investigate the usefulness of a spaceflight-relevant operational VR training environment as an effective countermeasure to spaceflight-induced neural decrements, we aim to quantify neural activation in brain regions of interest during task completion in such an environment. Our hypotheses were twofold: 1) that operational tasks will elicit increased brain activation compared to their microcognitive corollaries and 2) that the cortical regions activated will differ between the two VR training environments. Quantifying neural activation is an important first step to understanding the impacts of VR training and its potential applicability as a spaceflight-relevant countermeasure.

## 2 Methods

This study was completed under the University of Colorado Boulder IRB protocol number 23-0626. We recruited from a pool of healthy adults aged 18–65 years and disqualified potential subjects if they had a history of seizures, allergy to parabens (in EEG electrode gel), history of motion sickness [assessed as >90% on the Motion Sickness Susceptibility Questionnaire (Golding, 2006) or Visually Induced Motion Sickness Susceptibility Questionnaire (Keshavarz et al., 2023)], were colorblind, or had already trained in the rover operational environment (described below) through a previous study. All participants provided written informed consent to participate in the study.

### 2.1 VR environments

Two VR training environments were used for this study: an operational environment designed to capture key challenges of a Martian rover task, and a corollary environment designed to capture similar cognitive mechanisms but in a highly simplified, well validated laboratory task. Both environments were presented through a Meta Quest 2 VR head-mounted display (HMD) and completed with the use of a hand-held joystick (Logitech X52 HOTAS). The operational environment is an operationally relevant rover-based extravehicular activity (EVA) on the Martian surface. It includes three sequential subtasks which were each developed to match specific aspects relevant for planetary surface exploration and target specific cognitive domains of interest. The corollary environment is a series of three subtasks based on laboratory cognitive tests, each developed specifically to be a corollary version of the three operational subtasks by targeting the same cognitive domains as those used during the rover-based EVA. Table 1 outlines the three subtasks for each environment.

**Table 1:** Description and depiction of subtasks for operational and corollary environments.

Cognitive domains	Navigation, spatial awareness	Spatial planning, spatial reasoning	Visual search, spatial reasoning
Operational (Rover) Subtask	Rover Navigation (NAV)	Robot Arm (ARM)	Rock Observation (OBS)
	Navigating/driving a rover around a randomly generated field of obstacles on the Martian surface to reach a scientific point of interest. 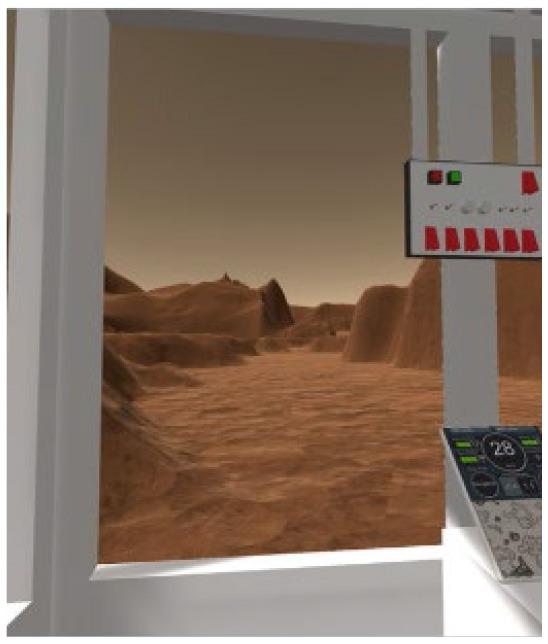	Operating a multi-degree-of-freedom robotic arm to repair an antenna cable by rotating and moving the cable end to a specific orientation and location. 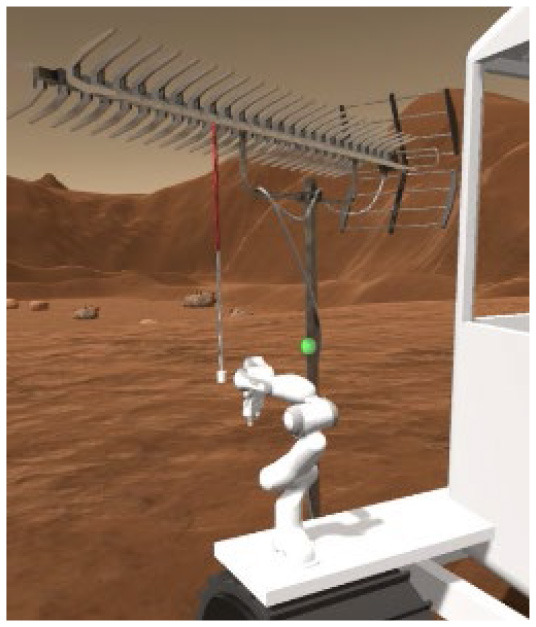	Visually searching through a field of rocks and identifying locations of rocks of scientific interest on 2D map. 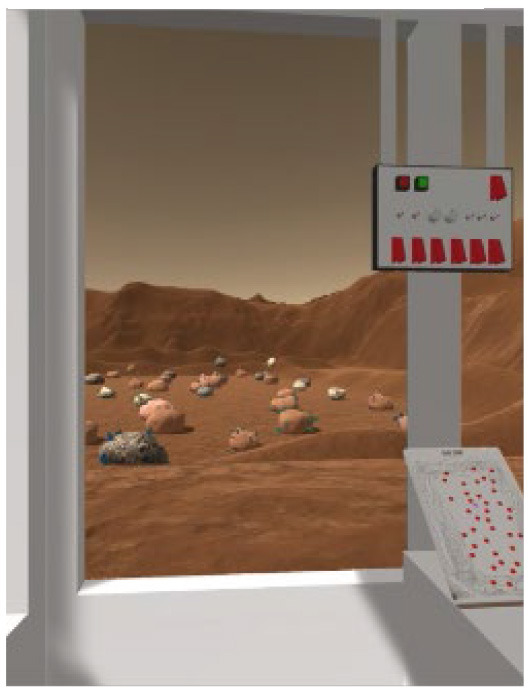
Matching Corollary Subtask	Triangle Completion Task (TCT)	Modified Mental Rotation (MMR)	Conjunctive Visual Search (VS)
	Drawing 2 sides of randomized triangle, given vertices, and completing triangle's last side without vertex information. 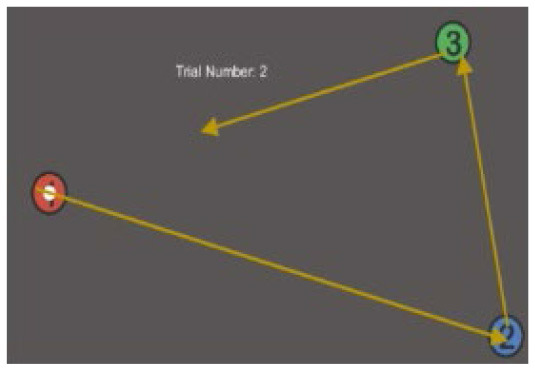	Rotating a 3-dimensional figure around 3 axes to match a target orientation. 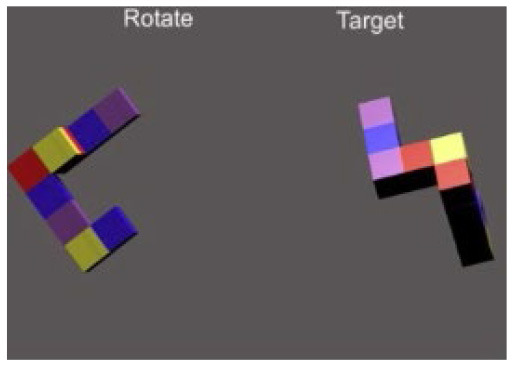	Visually searching 3x4 array of blue T's and red L's and indicating whether or not an array contains a red T. 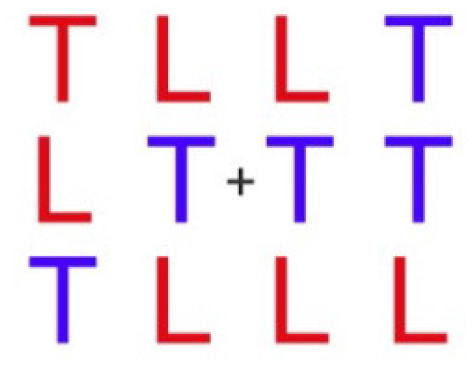

The following are the operationally relevant subtasks. The first subtask is a navigation (NAV) task that includes driving a rover around a randomly generated field of obstacles resembling the Martian surface. For this task, the rover's dashboard shows the user a map with their real-time location and the field of obstacles, as well as the scientific point of interest they must drive to through the field. The second of the operational subtasks is a robotic arm (ARM) task that involves operating a multi-degree-of-freedom robotic arm to repair an antenna cable that has become detached. The user must utilize their joystick to grasp the cable and move it to the plug to magnetically reattach it at a specific location and orientation, rotating and moving the robot end effector to do so. Finally, the last operational subtask is a rock observation (OBS) visual search task where the user must survey a field of rocks at the scientific location of interest they have driven to and utilize their joystick to mark locations of specific rocks of scientific interest (ROSIs) on a map on the rover dashboard. Table 1 displays visuals for all of these subtasks.

#### 2.1.1 Corollary tasks

To create the corollaries, we identified the general expected regions of cortical brain activation for each of the operational (rover) subtasks (NAV, ARM, and OBS) from previous studies that utilized similar ecological tasks. Corollary subtasks were then designed from the literature of common, validated laboratory tasks used to measure various aspects of cognitive performance, ensuring that they a) targeted the same previously identified cortical regions and b) could be implemented within a VR environment.

For the NAV subtask, the identified cortical regions of activation were the parietal cortex and pre-frontal cortex (PFC), which have been identified by various studies as the surface cortical regions most employed during navigation-focused ecological tasks (Bermudez-Contreras et al., 2020; Herweg and Kahana, 2018; Kober et al., 2013). The cognitive corollary selected was the Triangle Completion Task (TCT), commonly utilized to measure spatial orientation and navigation skill without the use of long-term memory. It had already been previously translated to VR (McLaren et al., 2022). We modified the task to be performed 2-dimensionally (i.e., completing the triangle from a bird's eye view above) to match the 2D map portion of the rover navigation task, with 5 trials completed before moving to the next.

For the ARM subtask, the primary expected regions of activation were the motor cortex, the PFC, and the parietal-occipital cortex, identified in studies that focused on the regions most employed in tasks requiring mental rotation and finer motor control (Cohen et al., 1996; Halari et al., 2006). The selected cognitive corollary was a Mental Rotation Task, which typically is used to measure spatial planning and reasoning by asking a subject to select the correct rotated version of a 3D shape from multiple options (Shepard and Metzler, 1971). To map this more closely to the operational ARM subtask, this corollary was modified to instead provide two of the same 3D shape: one to be rotated by the subject and one in a static target orientation that the subject must match (i.e., Modified Mental Rotation (MMR) task). Subjects completed 8 trials of this subtask before moving to the next subtask.

Finally, for the OBS subtask, the primary expected region of activation identified was the occipital cortex (as well as parts of the PFC), identified in studies that focus on cortical recruitment during visual observation tasks (Leonards et al., 2000; Nobre et al., 2003; Parker et al., 2014). As this operational subtask was based directly on a Conjunctive Visual Search (but made more complex), the corollary itself was a primed Conjunctive Visual Search (VS) task (Kristjánsson et al., 2002) of 40 trials, each with an array of letters in which a subject must identify whether or not there is a red T.

### 2.2 Protocol

Subjects each attended two sessions during the study. The first session served as a training session, during which they were familiarized with both VR training environments and subtasks. To start the first session, subjects filled out surveys with demographic information as well as caffeine/alcohol intake and sleep from the previous night.

Then, subjects were familiarized with the operational environment through an instructional video that provided all necessary information on how to use the joystick to complete all three subtasks and how they would be scored. After watching the video and reviewing key joystick interactions with an operator, subjects donned the VR HMD and were allowed up to 40 trials (with 1 trial being a completion of all 3 sequential subtasks) to reach the pre-established and validated level of proficiency in the operational environment. The rover operational environment uses an adaptive difficulty algorithm that begins training at level 12, developed in a previous study and determined to be the most effective regimen for VR training to achieve personalized skill acquisition for complex operational tasks (Verniani et al., 2024). This algorithm utilizes a 2-up, 1-down staircase and 24 discrete difficulty levels for each subtask, wherein performance per trial is categorized as ‘poor', ‘adequate', or ‘excellent' for each subtask. Subjects who did not reach proficiency at difficulty level 12 for all 3 subtasks in the allotted 40 trials were disqualified from the rest of the study (*n* = 6). We acknowledge that this potentially introduces selection bias toward individuals who are more adept at VR or complex spatial tasks. This may limit generalizability to a broader population, but likely remains applicable to astronauts in terms of being highly proficient in these types of operationally-relevant tasks. Those who did reach proficiency (*n* = 20, 10F/10M, age 24.9 + /−5.91 years) were retained as the final cohort of subjects.

After completing the operational training, subjects then watched a similar instructional video for the corollary environment. The corollary environment used static difficulty, meaning all training (and evaluation) was conducted at the same level of difficulty; all subjects completed 10 trials of training in the corollary environment for adequate familiarization.

The second session occurred between 18 and 48 h after the first session. Subjects donned a joint functional near-infrared spectroscopy (fNIRS) and electroencephalography (EEG) cap with a custom montage (see next section). The hardware was adjusted until high signal quality was achieved for each sensor system. Subjects were instructed to restrict movement to only what was task-necessary and to avoid talking during the data collection. Subjects donned the VR HMD for continuous data collection as they completed a block of 8 evaluation trials within both the corollary and operational environments, for a total of 16 trials over both environments. For the operational environment, difficulty was static (level 12) during the evaluation trials to ensure consistency. To balance any ordering effects, subjects were counterbalanced on which block environment (corollary or operational) they completed first, but all subjects completed both blocks with a brief 2-min break between blocks. Prior to each block, a 30s resting baseline was collected, and 5s pretrial baseline data were collected between each trial. A full evaluation trial lasted between 3–5 min for the operational environment and 2–4 min for the corollary environment.

### 2.3 Neuroimaging montage

**Figure 1:**
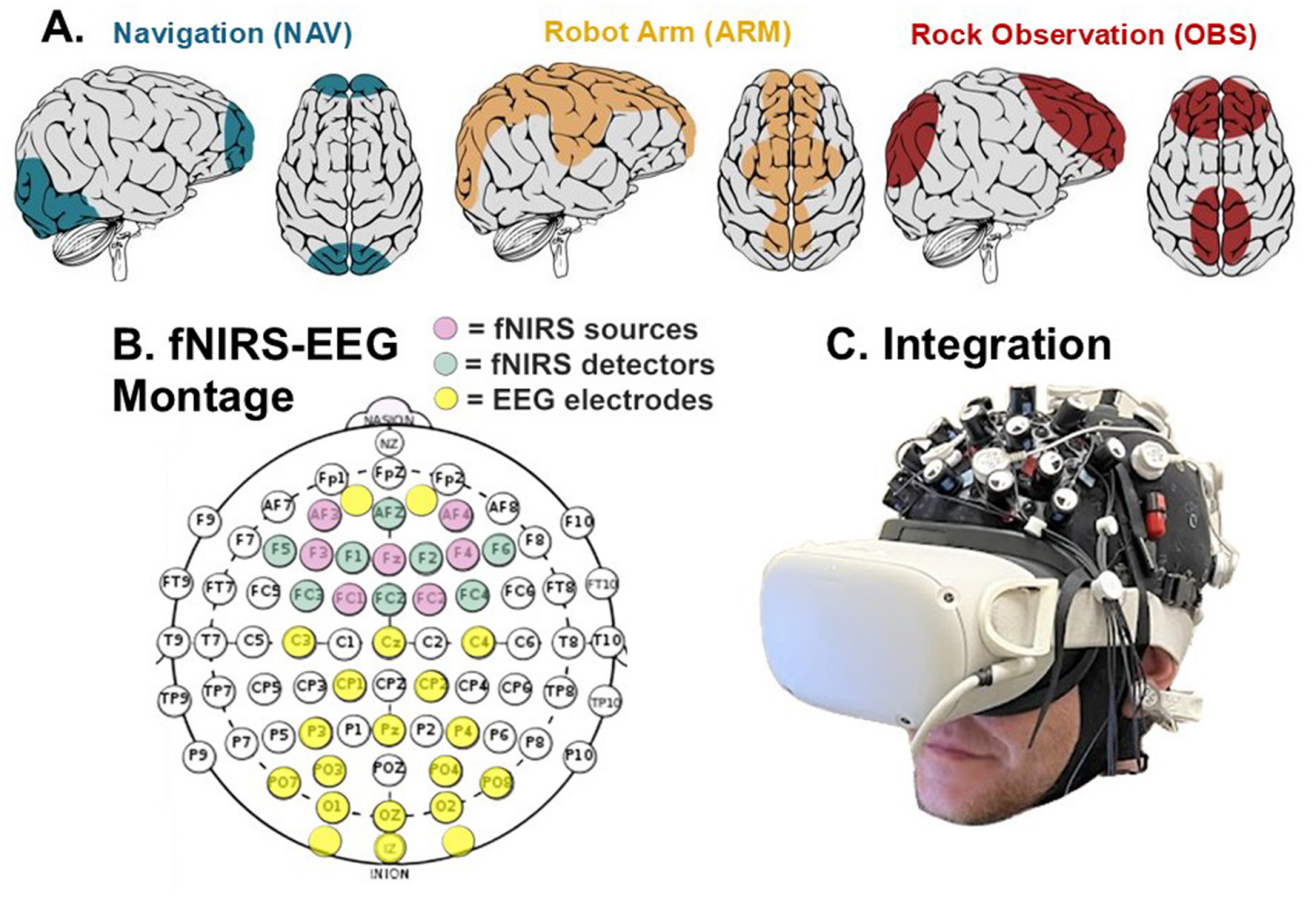
**(A)** Expected areas of activation for subtasks, **(B)** Joint fNIRS-EEG montage electrode and optode locations, **(C)** Montage-VR headset integration with custom strap system.

Based on the expected regions of activation for each operational (and subsequently corollary) subtask, we determined the specific overlying scalp locations to focus on for the study. These were then consolidated into three primary regions of interest, reflecting: the PFC, motor cortex, parietal cortex, and occipital cortex. The expected regions are shown topographically in Figure 1A.

A custom joint fNIRS and EEG montage was developed to capture neurophysiological activity in those primary regions of interest; this custom neuroimaging montage is shown in Figure 1B.

This study used a 20-channel EEG system (Enobio, Neuroelectrics) integrated into the same cap as a 20-channel fNIRS system (NIRsport2 7 source-8 detector, NIRx). As the aim was to study broad cortical activation rather than specific regional activation, the EEG was uniformly spread over the parietal, occipital and motor cortices, with a few electrodes capturing PFC activity, while the fNIRS optodes were focused on the frontal region where high signal quality and cortical activation is anticipated in each environment. EEG sample rate was 500 Hz, and fNIRS sample rate was 17 Hz.

These two neurophysiologic measures were used in conjunction with one another to allow for a more robust assessment of neurophysiological response, capturing both spatially resolute (fNIRS) and temporally resolute (EEG) data in tandem. Both measurement techniques are portable, noninvasive, and can be worn in the laboratory setting.

Montage development took into consideration areas on the head that may have interference from the VR HMD strap system and thus avoided including any electrodes or optodes on the frontopolar area (where the HMD rests against the forehead). In addition, a custom HMD strap system was developed to reduce the HMD strap's profile while maintaining a secure fit on the head. The resulting strap system, depicted in Figure 1C, was pilot tested to ensure it did not cause interference with data collection or cause notable subject discomfort.

### 2.4 Data preparation

#### 2.4.1 EEG

EEG data was cleaned using a non-causal 4-40 Hz second-order Butterworth IIR filter to remove movement artifacts (<4 Hz) and muscle artifacts (>40 Hz). An EEGLAB software-based channel rejection algorithm was used to reject poor channels (1.1 ± 0.69 removed per subject) and ASR (Artifact Space Reconstruction) algorithm was used to smooth over poor sections of data (considered as >20 standard deviations (SD) difference from the median of clean portions taken from the baseline) using Euclidian spline interpolation (Chang et al., 2018; Delorme and Makeig, 2004). Remaining extreme movement artifact spikes were identified using >20 SD difference from median data and removed using stable cubic spline interpolation (Roy and Shukla, 2017). EEGLAB-based Independent Component Analysis (ICA) was used to extract eye-blink, eye-movement, and heart artifact components, which were manually selected and removed (Delorme et al., 2007). This process was repeated for each subject.

Cleaned data was split using event markers at the beginning and end of each subtask. The 8 subtask trials were concatenated using spline interpolation between trials to avoid introducing discontinuity artifacts. Thus, each subject had 6 sets of concatenated data, 3 for the subtasks in the corollary task and 3 for the subtasks in the operationally relevant task. To reduce the variance-based error when comparing across datasets of variable lengths, the longer operational task datasets were shortened to match their corollary counterparts in length (though total lengths varied between subjects, they were the same between matched operational and corollary subtasks). This was done by combining sections from the beginning, middle, and end of the previously concatenated sets to form a new matched-length dataset, with spline interpolation once again being used to avoid introducing discontinuity/edge artifacts (Levy, 1987).

Spectral estimation was performed with Welch's periodogram method, which was run for each of the 6 concatenated datasets with a sampling frequency of 500 Hz and window size of 500 time points, with 50% overlap, for a frequency resolution of 1 Hz to provide the final power spectrum density (PSD) for each frequency band of interest (Ong et al., 2018). Data was analyzed by power in the alpha (8–12 Hz), theta (5–8 Hz), and low beta (12–20 Hz) bands.

**Figure 2:**
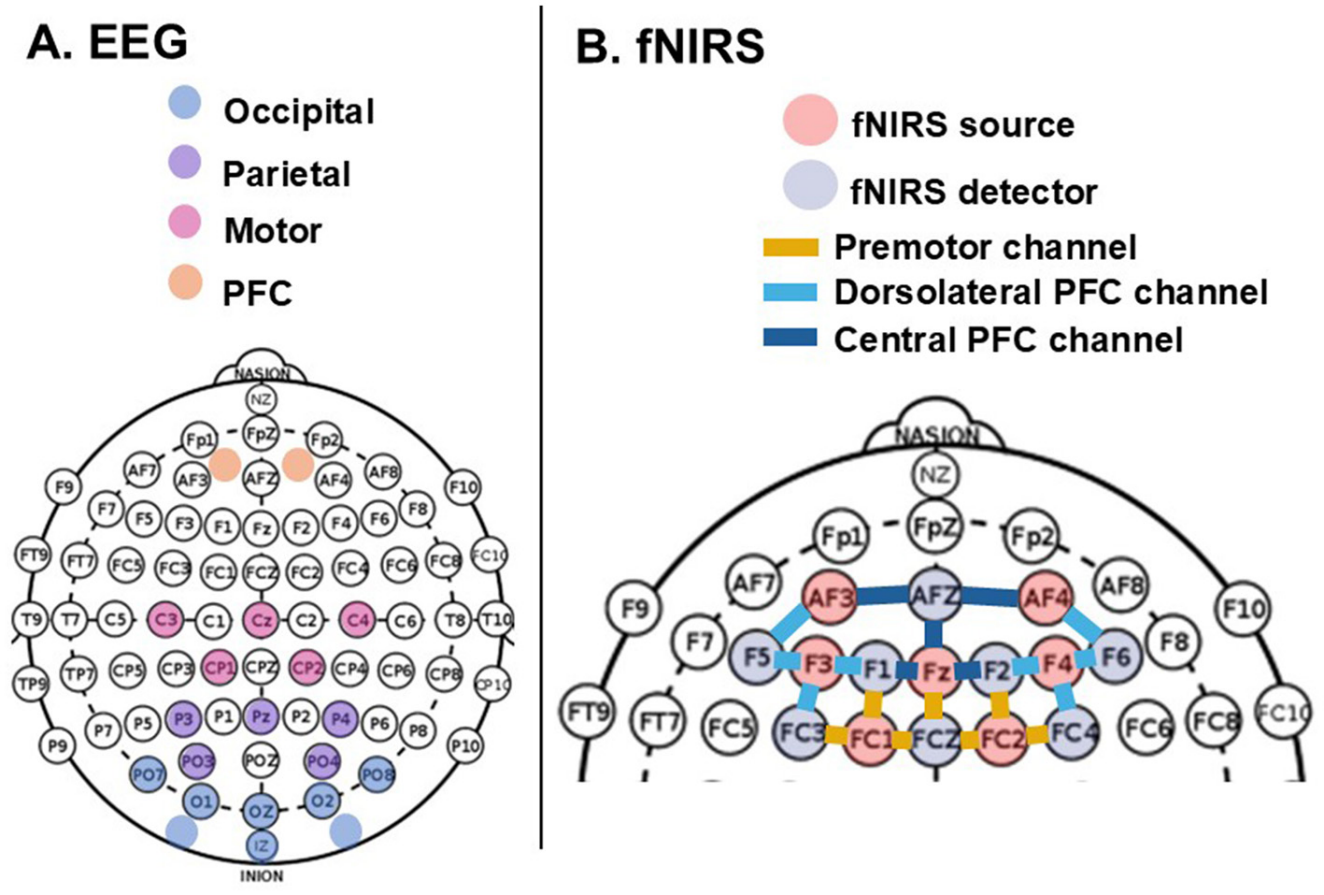
**(A)** EEG cortical regions and overlying channels determination, and **(B)** fNIRS PFC subregions and corresponding source-detector channels.

The EEG power was averaged into four channel systems overlaying cortical regions (depicted in Figure 2A). To determine whether a significant difference existed in activation level between corollary and operational conditions, PSD was averaged across subtasks for each condition, and the difference between the operational and corollary conditions, yielding a 3 x 4 matrix of mean PSD values for each subject (3 frequency bands x 4 overlaid cortical regions).

#### 2.5.2 fNIRS

fNIRS data was converted directly by the recording software from optical density to oxygenated hemoglobin (HbO) and deoxygenated hemoglobin (HbR) concentrations utilizing the modified Beer-Lambert Law. Poor-quality channels were visually inspected and manually removed according to manufacturer-given thresholds. The data was then cleaned with a 0.016–0.5 Hz bandpass filter to remove artifacts and shifted back by 6 s to account for the time delay associated with the hemodynamic response (Huppert et al., 2009). Remaining extreme movement artifact spikes were identified and removed using a common cubic spline interpolation approach (Cooper et al., 2012; Scholkmann et al., 2010). This process was repeated for each subject.

Cleaned data was sectioned the same way as EEG data, using event markers at the beginning and end of each subtask, but the eight different trials were retained separately. Thus, each subject had 8 sets of data for each of the 3 corollary subtasks and 3 operational subtasks. For each set of data and for each channel, peak amplitudes for ΔHbO and ΔHbR were extracted to characterize the ongoing hemodynamic response functions (HRF) (Barati et al., 2013; Kamran et al., 2016). The resulting peak amplitudes were averaged into three PFC subregions (outlined in Figure 2B) (Schommartz et al., 2021). To determine whether there was a significant difference between corollary and operational conditions, the peak amplitudes were averaged across subtasks for each condition, and the difference between the rover and corollary conditions was calculated, yielding a 3 x 2 x 8 matrix of peak amplitudes for changes in concentration of both the HbO and HbR chromophores for each subject (3 subregions x 2 chromophores x 8 trials).

### 2.5 Statistical analysis

Both EEG and fNIRS metrics satisfied the assumption of normality. Linear models were created for both mean PSD difference (between operational and corollary tasks) in EEG and mean peak amplitude in fNIRS. We used contrast coding to determine an overall grand mean (β_0_) and differences between regions and the overall mean (β_*i*_ for cortical region *i*, *r* = 3 regions for fNIRS, *r* = 4 for EEG). With contrast coding, the last regional difference from the overall mean is determined by the other coefficients and therefore dropped from the model. The fNIRS model included a subject-specific random effect *u*_*j*_ to account for idiosyncratic differences between subjects. The general forms of the EEG linear model and fNIRS linear mixed effects model (LMEM) are described in Equations 1, 2 respectively. In Equation 1, β_0_ is the global intercept representing the overall mean difference in EEG PSD between the operational and corollary tasks. In Equation 2, β_0_ is the mean difference in peak fNIRS amplitude between the operational and corollary tasks (i.e., a positive β_0_ indicates that the average EEG PSD or peak fNIRS amplitude averaged over all the regions is higher for the operational tasks than for the corollary condition). Three models were created for EEG, one for each frequency band, while two LMEMs were created for fNIRS: one for ΔHbO and one for ΔHbR.


$$\begin{array}{lll} Y_{i} & = & \;\beta_{0}+\sum_{r=1}^{3}\sum_{i=1}^{n}\beta_{r}R_{ir}+\epsilon_{i} \end{array}$$ (1)

$$\begin{array}{lll} Y_{ij} & = & \;\beta_{0}+\sum_{r=1}^{2}\beta_{r}R_{ijr}\;+u_{0j}+\epsilon_{ij}\;u_{0j}\;\sim \;N\left(0,\;\sigma \right). \end{array}$$ (2)

In addition, as the fNIRS data included trial-specific values, 20 separate linear regressions were conducted, 1 per fNIRS channel, to test for any ordering effect across trials, where a *t*-test was run with the null hypothesis that the slope of each regression was equal to 0. After Bonferroni multiple test corrections, there were no channels with significant ordering effects (and only two channels with unadjusted significance of *p* < 0.05), thus concluding that there is no statistically discernible order effect over the 8 experimental trials. Therefore, each of the 8 trials per subject was treated as independent and repeated measures in the LMEMs created using Equation 2.

For EEG, a one-sided *t*-test (α = 0.05) was run on the global intercept β_0_ for each frequency band against the null hypothesis that β_0_ = 0 (i.e., there is no difference in mean PSD between operational and corollary tasks). Significance levels were adjusted using a Bonferroni multiple tests correction to account for the 3 EEG models fit, so that significance was noted at *p* < 0.017.

For fNIRS, two one-sided *t*-tests (α = 0.05) were run on the global intercept β_0_ for the ΔHbO and ΔHbR mean peak amplitudes, with the null hypothesis that β_0_ = 0 (i.e., there is no difference in mean peak amplitudes between operational and corollary conditions). Significance levels were adjusted using a Bonferroni multiple tests correction, so that significance was noted at *p* < 0.025.

Finally, to determine whether there were differences between the region fixed effect coefficients β_*i*_, Tukey honest significant difference (HSD) multiple pairwise comparisons were run between regional coefficients for both EEG and fNIRS with a significance level of α = 0.05.

### 2.6 Exploratory analysis methods

The exploratory analysis aimed to determine whether matched subtasks between the rover operational and corollary environments might elicit activation in channels overlying the same regions of the brain. Due to the broad cortical coverage of the EEG electrodes and the centralization of the fNIRS channels on the PFC, only the EEG data was considered for this analysis. For this, the PSDs of the three frequency bands were not averaged across subtasks and channels overlying cortical regions, but rather averaged across subjects, plotted topographically for each of the 3 subtasks in each environment and compared between environments (using matched subtasks, Table 1) through visual inspection. As the large number of electrodes and variables would result in an extensive number of comparisons, and the analysis between the two environments was outside of the scope of this study, this analysis remained exclusively exploratory in nature, and should be considered descriptive and non-inferential.

## 3 Results

### 3.1 EEG

**Figure 3:**
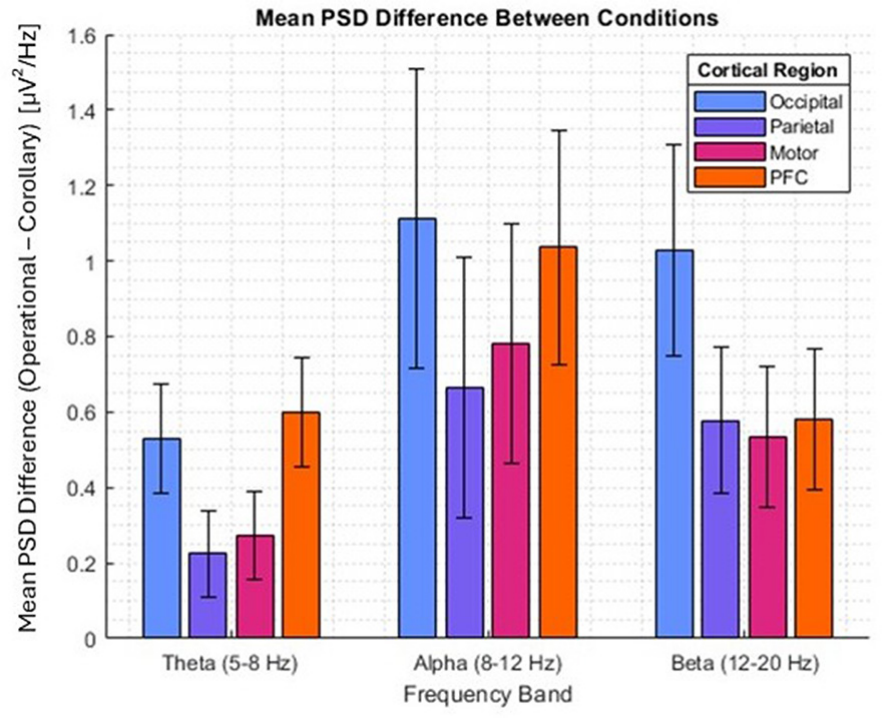
EEG mean PSD differences between operational and corollary conditions for theta, alpha, and beta frequency bands, segmented by channels overlying cortical region.

Figure 3 depicts the difference in EEG PSD between the operational (rover) and corollary conditions across the four cortical regions overlaid by EEG channels, where a positive y-value indicates higher activation within that frequency band in the operational tasks than the corollary tasks. Each EEG frequency band demonstrated statistically significant global intercepts (β_0, *theta*_ = 0.43, *p* < 0.0005; β_0, *alpha*_ = 0.83, *p* < 0.0005; and β_0, *beta*_ = 0.58, *p* < 0.0005). There were no statistical differences between region fixed effect coefficients.

### 3.2 fNIRS

**Figure 4:**
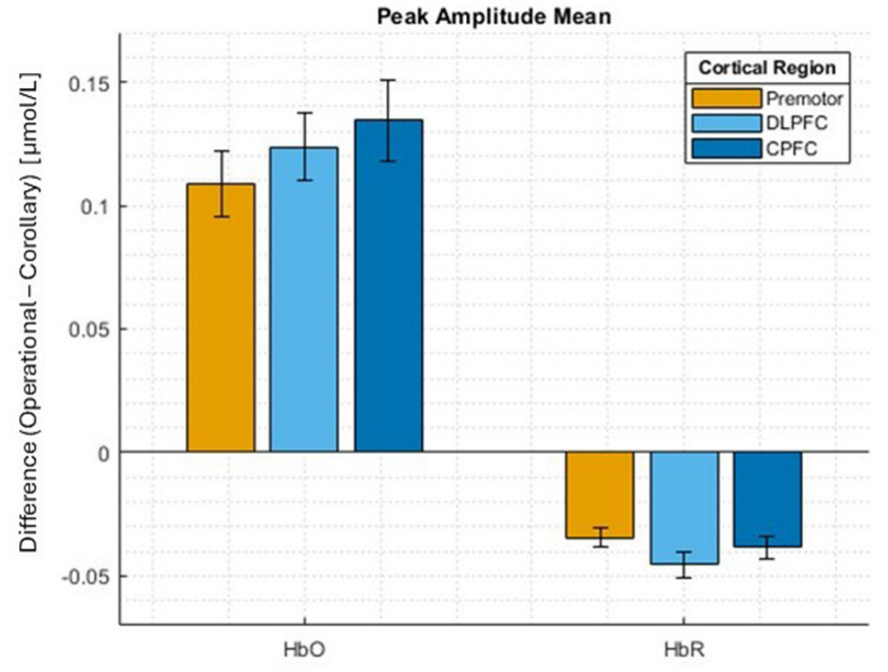
fNIRS ΔHbO and ΔHbR Mean peak amplitude differences between operational and corollary conditions, segmented by PFC subregion.

Figure 4 depicts the difference in both ΔHbO and ΔHbR mean peak amplitudes during HRF between the operational and corollary conditions across the four cortical regions. Both chromophores (i.e., ΔHbO and ΔHbR) demonstrated statistically significant global intercepts (β_0, *HbO*_ = 0.122, *p* < 0.001; β_0, *HbR*_ = −0.0394, *p* < 0.001). There were no statistical differences found between region fixed effect coefficients.

### 3.3 Exploratory analysis

**Figure 5:**
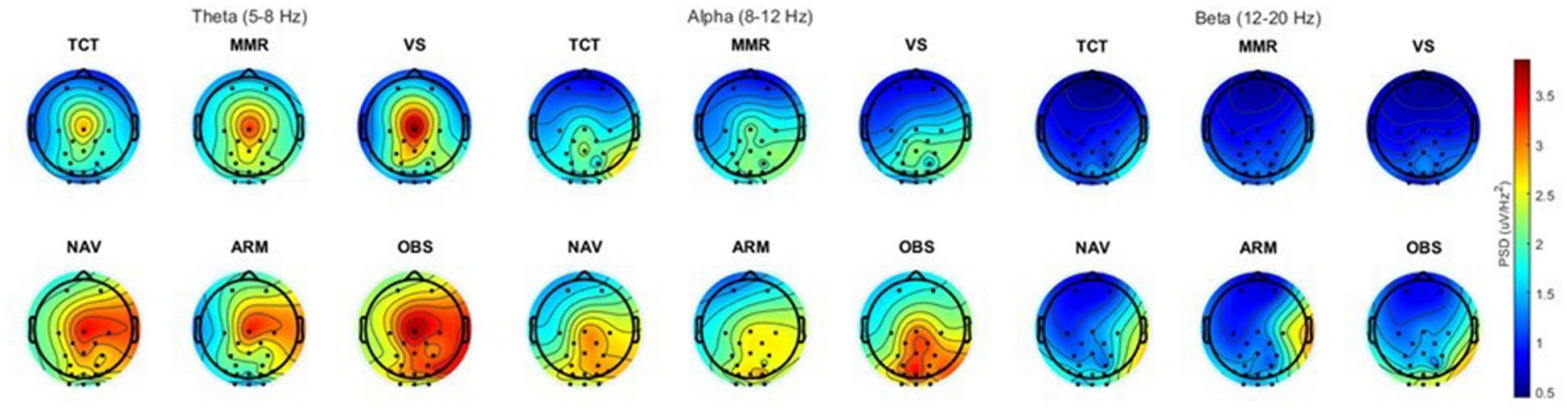
Interpolated topographical plots (nasion at top) of group EEG PSD means for each subtask, with the 3 corollary subtasks in the top row and the 3 operational subtasks in the bottom row.

Figure 5 depicts topography plots of mean PSD captured by EEG across all subjects in the three frequency bands and divided by subtask. In each band, there is little visible distinction between subtasks for both the corollary and operational tasks. However, there is visibly more activation in each frequency band during the operational subtasks than the corollary subtasks. In addition to this, there is visible consistency in regional activation within frequency bands across conditions, regardless of subtask.

## 4 Discussion

To begin to explore VR as a potential training countermeasure for spaceflight-associated neurological decrements, here we aimed to quantify the neural activation during LDEM-relevant operational tasks as compared to corollary tasks. Many previous studies have investigated how VR environments affect different aspects of cognition and neural activity (Dey et al., 2020; Mikropoulos, 2001; Ocklenburg and Peterburs, 2023), as well as how VR may be used in clinical rehabilitation settings (Chen et al., 2007; Feitosa et al., 2022; McEwen et al., 2014). However, previous work has largely focused on simpler environments and tasks or games, though some recent studies have expanded our understanding of neural activity in more complex VR environments, such as during interaction with virtual agents and training for surgery (Chang et al., 2022; De Melo et al., 2020; Natheir et al., 2023). Following this, we aim to inform a gap on cortical activity while completing complex tasks in an LDEM operationally-relevant VR environment.

Our results indicated that compared to cognitive-domain matched corollary tasks, the operationally relevant, high ecological validity tasks demonstrated increased brain activation. These increases were found to be agnostic to brain regions and frequency bands or chromophores analyzed. Further, in exploratory analyses, we found that these results tended to apply broadly across subtasks associated with the environments, and thus across cognitive domains, rather than being limited to specific subtasks or domains. In sum, our results suggest that operationally relevant tasks in VR produce enhanced neural activation as compared to simpler tasks, warranting further investigation as a potential countermeasure for spaceflight-associated neural decrements.

In EEG, the increase in power across three separate frequency bands for EEG for the more complex tasks is indicative of overall greater neural recruitment (Akin and Kiymik, 2000). Increases in alpha band power have been previously associated with increased memory load, processing in visual short-term memory, and processing of during visuospatial attention (Jensen, 2002; Johnson et al., 2011; Snyder and Foxe, 2010). Increases in theta band power have been associated with encoding of new information during the learning process and with increased cognitive effort during complex tasks (Dasari et al., 2017; Loonis et al., 2017; Zhu et al., 2021), while increases in beta band power have been associated with higher levels of attention and cognition (Singer, 1993; Wróbel, 2000). Interestingly, beta band power, especially in the motor cortex, is also well-known to decrease during execution of voluntary movement and increase during suppression of movement (Del Campo-Vera et al., 2020; Gola et al., 2013); given the nature of the experiment, it is possible that some of the beta band increases may be associated with requiring subjects to sit still while completing tasks. Further analysis, beyond the scope of this work, may reveal whether the overall increase in power across frequency bands is a broadband increase or due to differences in the balance of excitatory vs. inhibitory neural activity (Donoghue et al., 2020).

In addition to this, the fact that there were statistically significant simultaneous increases in each of the four systems of EEG channels overlaying different cortical regions suggests potential increased functional connectivity across regions during these tasks (Cole et al., 2021; Kelly and Castellanos, 2014), which may be relevant for maintenance of healthy neuroplasticity and overall cognitive function (Lövdén et al., 2013; Manganotti et al., 2022). While increased activation in operationally relevant tasks in VR may relate to beneficial effects, such as increased engagement, retention, or learning, we note that increased EEG power (or ΔHbO and ΔHbR responses) can also reflect fatigue, stress, compensatory processing, higher cognitive effort, or neural inefficiencies. We also note that the operationally-relevant tasks involve more complex visual stimuli, which naturally leads to more intensive processing of sensory input. While speculative, the broad increases in neural activity observed (across brain regions, frequency bands, and resulting across subtasks) may be indicative of multiple mechanisms, which should be investigated in the future, including mapping neural activation to performance, learning, or retention outcomes.

For fNIRS, increased neural activation is typically associated with a strong negative correlation in ΔHbO and ΔHbR during the hemodynamic response (Attwell and Iadecola, 2002; Friston et al., 1998). Furthermore, increases in ΔHbO in the PFC have been previously correlated with mental effort in flight-relevant operational contexts, with ΔHbO increases within the DLPFC specifically associated with higher cognitive workload while executing complex tasks (Ayaz et al., 2012; Causse et al., 2017). In our results, we found significantly larger ΔHbO peak amplitudes in the operational condition, as well as significantly negative differences in ΔHbR peak amplitudes, as compared to the corollary task. In addition to suggesting the operational environment was broadly more cortically engaging than the corollary, these outcomes also potentially indicate the operational subtasks as requiring more mental effort, which, if properly balanced, may help with improving neuroplasticity and cognitive function during LDEMs (Gkintoni et al., 2025; Smith et al., 2009).

While the subtask-specific exploratory analyses yielded little in terms of visible difference between the three different subtasks for both the operational and corollary environments, the clear consistency in terms of regional activation within frequency bands suggests a strong association between which regions of the brain the two environments recruit overall. Due to the large number of comparisons and scope of this analysis as secondary to our main focus, we did not run any statistical tests to validate the corollary subtasks as true cognitive domain matches for the operational subtasks; however, the consistency in measurements from overlying channels may serve as an indicator that the subtasks are properly designed to correspond with one another. Moreover, the visibly notable increase in power from the corollary to the operational subtasks when topographically plotted provides further evidence for the ability of the operational environment to elicit higher levels of broad neural activation than its corollary counterpart.

### 4.1 Limitations

While we explored the nature of power spectral density in EEG, the scope of this work did not cover time-frequency analyses or explore more complex EEG markers such as event-related potentials, event-related synchronization, or functional connectivity. This was largely due to the tasks being intrinsically complex and thus lacking specific stimuli, thus restricting our ability to analyze with a focus on EEG's high temporal resolution. The same was true for fNIRS, where the nature of the tasks and lack of specific stimuli created a more continuous HRF function rather than one associated with a certain stimulus onset.

Montage design was constrained by the joint mounting of a VR headset, and despite comfort-testing the strap system design and limiting the duration of the time spent in all of the neuroimaging equipment and VR, many subjects self-reported fatigue and onset of headache symptoms that were alleviated only after the equipment was removed. It is important to note that the symptoms reported are also common signs of VR-related motion sickness and Oculus Meta Quest wear; the effect of these symptoms on subject performance and cognitive function cannot be fully characterized. While not incorporated into our analysis, as reports were subjective and not systematically collected, the contributions of wearing the physical VR headset and EEG-fNIRS cap were presumably similar between the operationally-relevant and corollary tasks.

### 4.2 Future work

In this study, both EEG and fNIRS markers demonstrated the utility of a complex VR environment comprised of operationally-relevant tasks for stimulating broad cortical activation. It is thought that the structural and functional neurological changes that occur during long duration spaceflight are to some degree associated with the limited and altered stimulation environment in the isolated and confined environment. However, future work is needed to understand the level to which these forms of stimulation may help counter the neurological changes that occur in space, so as to inform the utility of similar VR trainings as an on-orbit or in-transit countermeasure. To this end, future studies should focus on exploring functional connectivity and coherence between different cortical regions during these operational VR tasks.

Further analyses looking at task performance may provide insight into performance correlations with neural activity, as well as any associations between performance, neural activity, and previous VR experience, which varied widely among this study's subjects, to help inform the utility of extensive experience with VR in astronaut training. Finally, future work should aim to determine the precise neural mechanisms of how VR stimulates the brain and whether targeting specific cognitive domains may aid in avoiding neural degradation during spaceflight.

## 5 Conclusion

This study provides a basis to understand how VR simulations with operationally-relevant tasks engage and elicit activation within different regions of the brain. The cognitive and neural changes seen through spaceflight are numerous, and a current lack in knowledge of the mechanisms behind them makes it difficult to design countermeasures for these degradations. VR training environments have been used extensively for cognitive rehabilitation through entraining various regions of the brain, and this, paired with the versatility, cost efficacy, and simplicity of VR renders it a feasible and appealing future countermeasure during spaceflight. However, there is a distinct lack of research and understanding of how complex, immersive VR environments with operationally relevant tasks may recruit regions of the brain as compared to microcognitive tasks, which are currently commonly used to assess astronaut cognitive function and performance during spaceflight. By comparing this to a reference of microcognitive-based corollary tasks designed to match the targeted cognitive domains, we begin to quantify the neural effects of a complex task-based VR environment in eliciting increased brain function. With VR already on the forefront of astronaut training research, this serves to illustrate another of its potential uses as a countermeasure, though further work is still needed to understand how exactly VR's entrainment of neural processes can counter the structural and cognitive decrements seen in long-duration spaceflight.

## Data Availability

The raw data supporting the conclusions of this article will be made available by the authors, without undue reservation.
